# Barriers and enablers for implementation of digital-linked diagnostics models at point-of-care in South Africa: stakeholder engagement

**DOI:** 10.1186/s12913-024-10691-z

**Published:** 2024-02-16

**Authors:** Boitumelo Moetlhoa, Siphesihle R. Nxele, Kuhlula Maluleke, Evans Mathebula, Musa Marange, Maureen Chilufya, Tafadzwa Dzinamarira, Evans Duah, Matthias Dzobo, Mable Kekana, Ziningi Jaya, Lehana Thabane, Thobeka Dlangalala, Peter S. Nyasulu, Khumbulani Hlongwana, Thembelihle Dlungwane, Mankgopo Kgatle, Nobuhle Gxekea, Tivani Mashamba-Thompson

**Affiliations:** 1 School of Health Systems and Public Health, Faculty of Health Sciences, University of Pretoria, Pretoria, South Africa; 2 Medical and Scientific Affairs, Infectious Diseases Emerging Markets, Rapid Diagnostics, Abbot Rapid Diagnostics (Pty) Ltd, Johannesburg, South Africa; 3 Department of Health Research Methods, Evidence, and Impact, McMaster University, Hamilton, Canada; 4 Department of Radiography, Faculty of Health Sciences, University of Pretoria, Pretoria, South Africa; 5 Department of Global Health, Stellenbosch University, Stellenbosch, South Africa; 6 Department of Public Health Medicine, University of KwaZulu-Natal, Durban, South Africa; 7 School of Nursing and Public Health, University of KwaZulu-Natal, Durban, South Africa; 8 Nuclear Medicine Research Infrastructure, University of Pretoria, Pretoria, South Africa

## Abstract

The integration of digital technologies holds significant promise in enhancing accessibility to disease diagnosis and treatment at point-of-care (POC) settings. Effective implementation of such interventions necessitates comprehensive stakeholder engagements. This study presents the outcomes of a workshop conducted with key stakeholders, aiming to discern barriers and enablers in implementing digital-connected POC diagnostic models in South Africa. The workshop, a component of the 2022 REASSURED Diagnostics symposium, employed the nominal group technique (NGT) and comprised two phases: Phase 1 focused on identifying barriers, while Phase 2 centered on enablers for the implementation of digital-linked POC diagnostic models. Stakeholders identified limited connectivity, restricted offline functionality, and challenges related to load shedding or rolling electricity blackouts as primary barriers. Conversely, ease of use, subsidies provided by the National Health Insurance, and 24-h assistance emerged as crucial enablers for the implementation of digital-linked POC diagnostic models. The NGT workshop proved to be an effective platform for elucidating key barriers and enablers in implementing digital-linked POC diagnostic models. Subsequent research endeavors should concentrate on identifying optimal strategies for implementing these advanced diagnostic models in underserved populations.

## Introduction

The digital age represents a transformative economic and social phenomenon driven by key technologies, including artificial intelligence, the Internet of Things (IoT), nanotechnology, biotechnology, and robotics [1]. The emergence of digital technologies has significantly impacted efficiency, effectiveness, and reduced healthcare service costs [2, 3]. These technologies enable remote connectivity between patients and healthcare providers, expanding access to diagnostics, treatment, and follow-up care [3].

Digital technologies leverage algorithms and data analytics, presenting potential benefits to healthcare systems. A critical consideration, particularly in South Africa, is their capacity to surmount language barriers. Within healthcare settings, language barriers frequently give rise to miscommunications between health professionals and patients [4], consequently diminishing the quality of healthcare delivery. This can lead to increased mismanagement, jeopardize patient safety through inadequate assessment, misdiagnosis, and delayed treatment. As a consequence, patients may develop diminished confidence in healthcare systems [4, 5]. Recognizing this challenge, digital technologies have the capability to integrate translation applications, enhancing interactions between healthcare providers and patients who speak diverse languages [3]. In developing countries, including those facing a shortage of healthcare specialists, digital technologies and machine learning play a pivotal role in fortifying disease management systems within the health sector [3, 6]. Moreover, machine learning has the potential to augment the predictive capabilities of non-expert physicians, leading to heightened accuracy in the diagnosis and treatment of health conditions [3, 7].

The advent of the digital technologies has given rise to intelligent environments by integrating the Internet of Things (IoT) and smartphones into imaging, sensing, and diagnostic services. Point-of-Care (POC) tests equipped with Bluetooth Low Energy (BLE) technology facilitate data connectivity over short distances, exemplified by innovative devices like the non-invasive mouth guard biosensor for monitoring salivary glucose and fitness trackers designed to track patients' physical activity and vital signs [8]. Additionally, digital diagnostic devices offer the capability to analyze various non-invasive samples, including sweat, saliva, feces, tears, and breath, to detect biomarkers associated with major diseases such as cancer and HIV [9].

The implementation of these digital-linked Point-of-Care (POC) diagnostic models has demonstrated significant benefits in developed countries and holds potential for enhancing healthcare systems in underserved communities. However, there is currently limited evidence regarding the deployment of such POC diagnostic models in low- and middle-income countries, including South Africa. Therefore, this study aims to identify the barriers and enablers influencing the implementation of digital-linked POC diagnostic models in South Africa. In this investigation, barriers are delineated as factors that may impede the implementation of these models, while enablers are characterized as factors that have the potential to facilitate and attract the implementation of digital-linked POC diagnostic models.

## Methods

Key stakeholders actively engaged in the 1st REASSURED Diagnostics symposium and workshop, organized by the *REASSURED-d@UP* research group in November 2022. Stakeholders were identified as individuals possessing expert knowledge in POC diagnostics, expressing perspectives on digital-linked diagnostic models, and demonstrating a vested interest in the sustainable implementation of POC diagnostics within healthcare systems.

### Study participants and sampling

Invitation letters were sent via email to key stakeholders of POC diagnostic tests internationally, subject specialists, researchers, and diagnostic test developers.

#### Inclusion criteria

The study included people who fulfilled the following criteria:

- Researchers whose studies are focused on POC diagnostic tests
- Academics who are POC diagnostic test subject specialists
- Diagnostic test development entities involved in developing, manufacturing, and commercializing POC diagnostic tests
- Individuals who would share their view on incorporating digital-linked POC diagnostics
- Individuals who are able to communicate in the English language

#### Exclusion criteria

The study excluded individuals based on the following criteria:

- Personnel who have no expertise in POC diagnostic test research and development
- Individuals who did not give consent to participate in the study

### Workshop program

Data were collected during a nominal group technique (NGT) workshop [10, 11] held on November 23, 2022. We explored participants’ perceptions of barriers and enablers of the implementation of digital-linked POC diagnostic models in South Africa. The workshop was conducted in two phases. Phase 1 and phase 2 focused on determining the barriers and enablers, respectively, of the implementation of digital-linked POC diagnostic models in South Africa. BM, SRN and TMT facilitated the workshop.

#### Phase one

The PI (BM) introduced the workshop to the key stakeholders, and participants were divided into two groups. The PI posed the question: *What are the barriers for implementation of digital-linked POC diagnostic models in South Africa*? Following the instructions from the facilitators, stakeholders listed their suggestions of barriers into themes. The PI listed the themes in a form on google docs to enable voting through ranking. Participants then completed the google form. The emerging ideas or themes were ranked by assigning a value to a theme according to its priority. The themes were ranked using a Likert scale from 1–7, with one representing a very low priority and seven representing the highest priority.

#### Phase two

The PI (BM) posed the question: *What are the enablers for implementation of digital-linked POC diagnostic models in South Africa*? Stakeholders listed their suggestions of enablers into themes. The PI listed the themes in a form on google docs to enable voting through ranking. Participants then completed the google form. The emerging ideas or themes were ranked by assigning a theme to an idea according to its priority. The themes were ranked using a Likert scale from 1–7, with one representing a very low priority and seven representing the highest priority.

Following the NGT workshop, a report presenting the results was compiled by BM and shared with the key stakeholders for comments.

## Results

Eighteen key stakeholders agreed to participate in the workshop. The stakeholders were predominately women (60%). Most (65%) of the participants were employed while 35% were full-time postgraduate students. Characteristics of participants are presented in Table 1.

**Table 1:** Characteristics of workshop participants

Gender (Male/Female/Trans/Non-binary)	Occupation
Male	Qualitative research expert
Female	Point-of-care diagnostics researcher
Male	Qualitative research expert
Female	Public health education expert
Female	Point-of-care diagnostics researcher
Male	Diagnostic developer
Male	Diagnostic developer
Male	Point-of-care diagnostics researcher
Female	Medical student
Female	Medical scientist
Male	Medical scientist
Male	Point-of-care diagnostics researcher
Female	Point-of-care diagnostics researcher
Female	Point-of-care diagnostics researcher
Female	Point-of-care diagnostics researcher
Female	Point-of-care diagnostics researcher
Female	Point-of-care diagnostics user
Female	Point-of-care diagnostics user
Female	Point-of-care diagnostics user

**Fig. 1:**
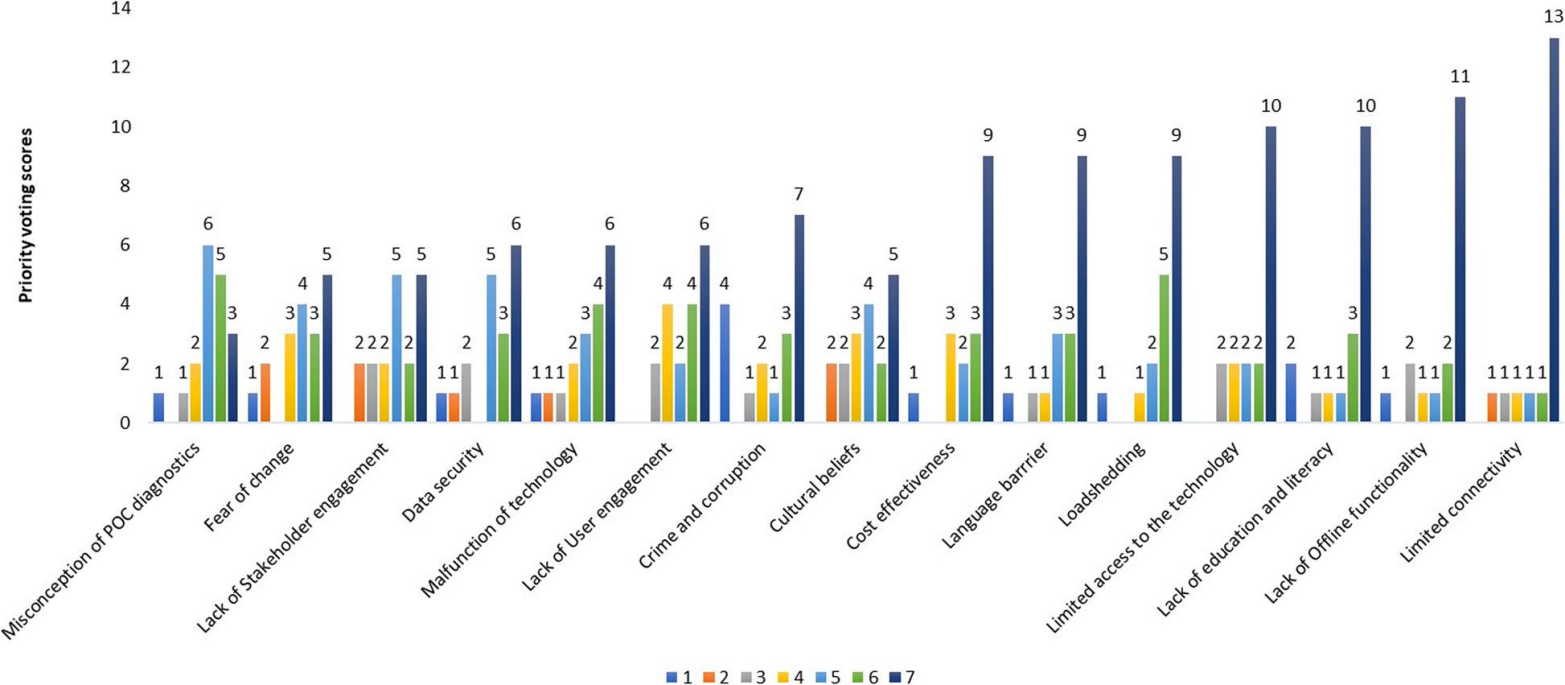
Key stakeholders’ perception of barriers to implementing digital-linked point-of-care models in South Africa. The results are shown on a Likert scale with one being the lowest priority and seven the highest priority

Stakeholders reported 15 factors as barriers to implementing digital-linked POC models in South Africa (Fig. 1). The Likert scale voting results showed that connectivity was considered a major barrier with 13 participants giving it a score of seven (high priority). Participants also identified offline functionality (*n* = 11), limited access to the technology (*n* = 10), lack of education and literacy (*n* = 10), load shedding (*n* = 9), language barriers (*n* = 9), cost effectiveness (*n* = 9), crime and corruption (*n* = 7), lack of user engagement (*n* = 6), malfunction of technology (*n* = 6) lack of data security (*n* = 6), lack of stakeholder engagement (*n* = 5), fear of change (*n* = 5), and cultural beliefs (*n* = 5) as being high priority barriers. Misconception of POC diagnostics was identified as the least important barrier with only three participants scoring it as high priority.

**Fig. 2:**
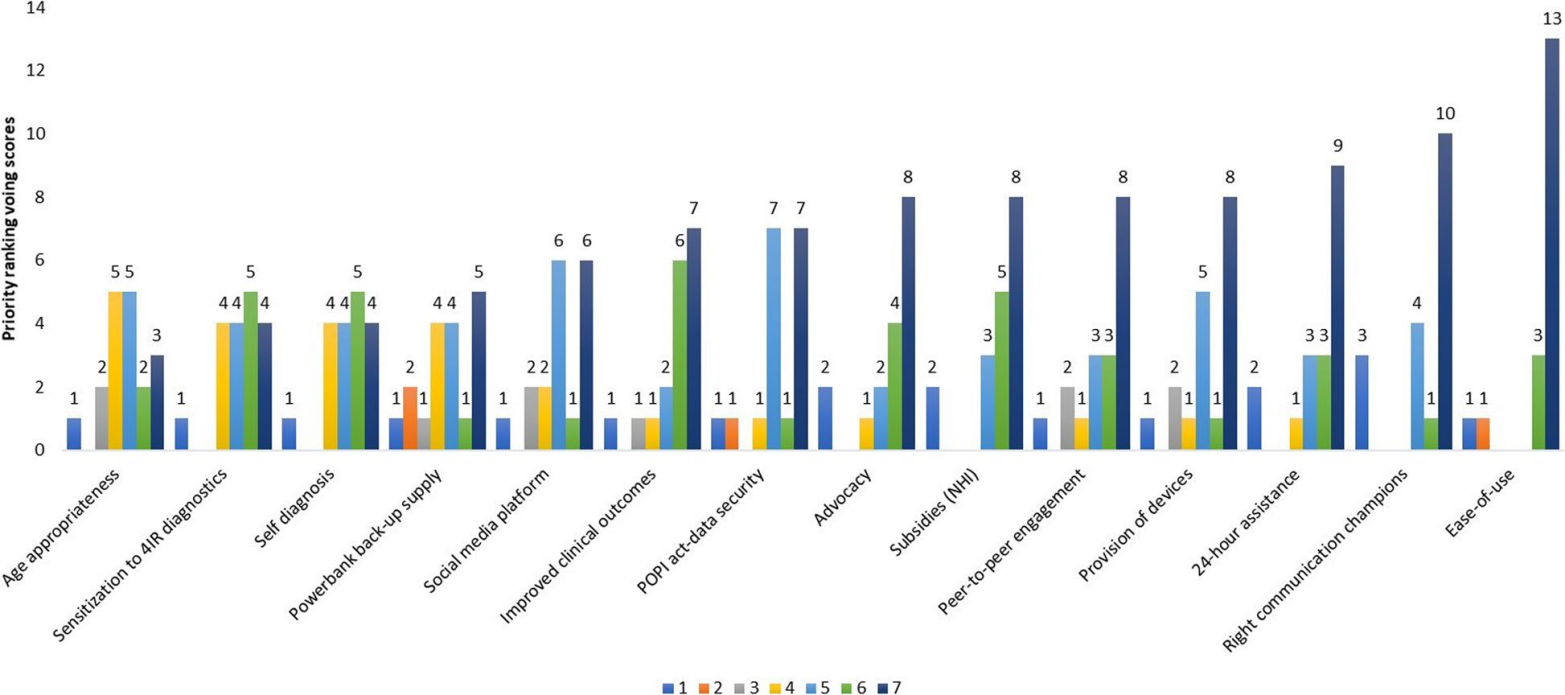
Key stakeholders’ perception of enablers to implementing digital-linked point-of-care models in South Africa. The results are shown on a Likert scale with one being the lowest priority and seven the highest priority

Stakeholders further identified 14 factors that would enable the implementation of digital-linked POC models in South Africa (Fig. 2). Thirteen participants scored ease of use as being a high priority enabler. Other high priority enablers included accurate communication champions (*n* = 10), 24-h assistance (*n* = 9), provision of devices (*n* = 8), peer-to-peer engagement (*n* = 8), National Health Insurance (NHI) subsidies (*n* = 8), advocacy (*n* = 8), Protection of Personal Information (POPI) Act-data security (*n* = 8), improved clinical outcomes (*n* = 7), social media platform (*n* = 6), power bank back-up supply (*n* = 5), self-diagnosis (*n* = 4) and sensitization to digital diagnostics (*n* = 4). Age appropriateness was the enabler with the fewest least high priority scores (*n* = 3).

To show the relative importance of barriers, we summed the scores given for each barrier and then ranked the total scores from lowest to highest (Table 2). Limited connectivity was the most important barrier (88.1%) followed by load shedding (85.7%), lack of offline functionality (84.1%), limited access to the technology (84.1%), language barrier (82.5%), cost effectiveness (82.5%), and lack of education and literacy (81%).

**Table 2:** Relative importance of barriers to implementing digital-linked point-of-care models in South Africa

**Barriers to implementing digital-linked POC models**	**Score** *1* = *low priority* *7* = *high priority*							**Total**	**Percentage rank**
	**1**	**2**	**3**	**4**	**5**	**6**	**7**	**126**	**100%**
Crime and corruption	4		1	2	1	3	7	87	69
User engagement			2	4	2	4	6	88	70
Cultural beliefs		2	2	3	4	2	5	89	70.6
Fear of change	1	2		3	4	3	5	90	71.4
Stakeholder engagement		2	2	2	5	2	5	90	71.4
Misconception of POC diagnostics	1		1	2	6	5	3	93	73.8
Data security	1	1	2		5	3	6	94	74.6
Malfunction of technology	1	1	1	2	3	4	6	95	75.4
**Education and literacy**	**2**		**1**	**1**	**1**	**3**	**10**	**102**	**81.0**
**Cost effectiveness**	**1**			**3**	**2**	**3**	**9**	**104**	**82.5**
**Language barrier**	**1**		**1**	**1**	**3**	**3**	**9**	**104**	**82.5**
**Access to the technology**			**2**	**2**	**2**	**2**	**10**	**106**	**84.1**
**Offline functionality**	**1**		**2**	**1**	**1**	**2**	**11**	**106**	**84.1**
**Load shedding**	**1**			**1**	**2**	**5**	**9**	**108**	**85.7**
**Connectivity**		**1**	**1**	**1**	**1**	**1**	**13**	**111**	**88.1**

To show the relative importance of enablers, we summed the scores given for each enabler and then ranked the total scores from lowest to highest (Table 3). The most important enabler was ease of use (88.9%) followed by improved clinical outcomes (81.7%), NHI subsidies (81.7%), 24-h assistance (81%), and peer-to-peer engagement (79.4%).

**Table 3:** Relative importance of enablers of implementing digital-linked point-of-care models in South Africa

**Enablers to implementing digital-linked POC models**	**Score** *1* = *low priority* *7* = *highly priority*							**Total**	**Percentage rank**
	**1**	**2**	**3**	**4**	**5**	**6**	**7**	**126**	**100%**
Age appropriateness	1		2	5	5	2	3	85	67.5
Back-up supply-power banks	1	2	1	4	4	1	5	89	70.6
Social media platform	1		2	2	6	1	6	93	73.8
Self-diagnosis	1	1			9	1	6	96	76.2
Advocacy	2			1	2	4	8	96	76.2
POPI Act-data security	1	1		1	7	1	7	97	77
Provision of devices	1		2	1	5	1	8	98	77.8
Sensitization to digital diagnostics	1			4	4	5	4	99	78.6
Right communication champions	3				4	1	10	99	78.6
**Peer-to-peer engagement**	**1**		**2**	**1**	**3**	**3**	**8**	**100**	**79.4**
**24-h assistance**	**2**			**1**	**3**	**3**	**9**	**102**	**81**
**NHI subsidies**	**2**				**3**	**5**	**8**	**103**	**81.7**
**Improved clinical outcomes**	**1**		**1**	**1**	**2**	**6**	**7**	**103**	**81.7**
**Ease of use**	**1**	**1**				**3**	**13**	**112**	**88.9**

### Stakeholder feedback

We sent the results of the workshop to all 18 participants and asked them to comment on the barriers and enablers that were identified during the workshop. Three participants gave feedback and provided their opinion on the impact of connectivity, load shedding, and access to technology as important barriers to the implementation of digital-linked POC diagnostic models.

Key stakeholders prioritized connectivity as a barrier with negative implications on the utility of the digital-linked POC devices: “*I believe this is a major problem. Majority of the population in South Africa is in what we call remote areas that often do not have cellular phone network*.”

“*With poor connectivity, the devices become unusable*.”

Load shedding, or rolling electricity blackouts, was another important barrier that the participants gave their opinion on: “*The impact of load shedding is huge. Without electricity, devices cannot be charged when out of battery and networks are disturbed with load shedding; Load shedding presents many ripple effects on POC; Inability to access databases among other challenges.*”

“*It is quite significant as most of these devices have to be charged. However, in terms of severity, it may not be the most severe as often there are phases of load shedding and power is available at some point on each day.*”

“*Most of these products run on electricity and power cuts render them ineffective.*”

Lastly, limited access to technology is an important barrier as it can negatively affect the implementation of digital-linked POC diagnostic models: “*As of July 2022, an estimated 22 million people in South Africa used a smartphone, which accounts for about one third of the country's population. This means two thirds still do not have access to this technology. This population is at risk of being left out in the digital technology where results can be virtually shared with health specialists and advisors*.”

Participants also discussed the most important enablers of digital-linked POC diagnostic model implementation. Participants stressed that that the technology had to be easy to use and that there should be 24-h assistance. Devices have to be easy to use because “*this enables effective use by people of all status particularly, the non-elite. This defeats inequality.”*

**“***The simpler the technology the greater the uptake. Complex technology is likely to discourage uptake.”*

Participants also explained that 24-h assistance would be an important enabler: *“This is towards improvement of product functionality and to facilitate ease-of-use.”*

## Discussion

We present the findings from an engagement session with key stakeholders that enabled the identification of crucial barriers and enablers for implementing digital-linked POC diagnostic models in South Africa. Similar to other settings in SSA, South Africa faces distinctive challenges, leading to significant barriers in the implementation of digital-linked POC diagnostic models. These challenges include limited connectivity, instances of load shedding, and restricted access to technology. Addressing these barriers is imperative, necessitating policymakers responsible for device selection to ensure that these technologies are user-friendly, contribute to enhanced clinical outcomes, and are supported by 24-h assistance.

South Africa has grappled with an energy crisis, marked by persistent rolling electricity blackouts or load shedding since 2007. The current situation reflects the highest recorded load shedding hours, averaging over 1500 h since its inception [12]. ESKOM (Electricity Supply Commission) remains the exclusive electricity supplier, and challenges in power supply are anticipated to persist [13]. Load shedding emerges as a major obstacle, impeding the country's ability to embrace and effectively utilize new technologies [13]. Access to technology poses another significant barrier, encompassing communication support, process structuring, and information processing inherent in technologies such as artificial intelligence, the Internet of Things (IoT), and blockchain [13, 14].

Blockchain, as a technological tool ensuring transparency in transactions of verified data over networks with minimal third-party involvement, integrates elements like the internet, cloud computing, IoT, big data, and cybersecurity. Its potential impact spans eHealth, smart energies, advanced manufacturing, and more [14, 15]. South Africa, noted for low levels of technological development and momentum, appears unprepared for the adoption of a digital economy [16]. Within the healthcare sector, Africa as a continent grapples with fragmentation, silos, and an inability to integrate healthcare information, restricting participation in digital-linked healthcare systems [16]. The country's limited access to digital technology impedes progress in technological advancements, especially in rural areas disproportionately affected by load shedding and connectivity issues. Rural communities in South Africa lag in efficient healthcare provision due to insufficient funding, inadequate technological infrastructure, and inadequately trained healthcare workers [17]. Digital-linked technologies hold the potential to enhance healthcare quality in rural South Africa [17], addressing the barriers identified in this study will necessitate significant investment.

In summary, a collaborative effort among key stakeholders transpired during a NGT workshop, facilitating the collective identification of paramount barriers and enablers in implementing digital-linked POC diagnostic models in South Africa. A notable limitation of this study was the underrepresentation of POC diagnostic test users relative to experts and researchers, which restricts the generalizability of the findings to broader POC diagnostic test user populations. Nevertheless, this approach fostered the co-creation of pivotal factors that must be considered for the successful implementation of digital-linked POC diagnostic models in South Africa. We advocate for an intervention study aimed at delineating optimal strategies for surmounting barriers to implementing digital-linked POC models, especially in rural communities in South Africa.

## Data Availability

All data analyzed in the study is available upon written request from the corresponding author.
